# Telemedicine as an Option for Monitoring Metabolic Dysfunction-Associated Fatty Liver Disease (MAFLD) Patients Facing the COVID-19 Pandemic: A Systematic Review and Meta-Analysis

**DOI:** 10.3390/jpm14030281

**Published:** 2024-03-02

**Authors:** Femmy Nurul Akbar, Safira Rosiana Choirida, Ahmad Zaqi Muttaqin, Fika Ekayanti, Hoirun Nisa, Hari Hendarto

**Affiliations:** 1 Department of Internal Medicine, Faculty of Medicine, Universitas Islam Negeri Syarif Hidayatullah Jakarta, South Tangerang 15419, Banten, Indonesia; safira.rosiana16@mhs.uinjkt.ac.id (S.R.C.); ahmad.zaqi16@mhs.uinjkt.ac.id (A.Z.M.); hari.hendarto@uinjkt.ac.id (H.H.); 2 Department of Community Medicine, Faculty of Medicine, Universitas Islam Negeri Syarif Hidayatullah Jakarta, South Tangerang 15419, Banten, Indonesia; fikaekayanti@uinjkt.ac.id; 3 Department of Public Health, Faculty of Health Sciences, Universitas Islam Negeri Syarif Hidayatullah Jakarta, South Tangerang 15419, Banten, Indonesia; hoirun.nisa@uinjkt.ac.id

**Keywords:** telemedicine, metabolic dysfunction-associated fatty liver disease, monitor, lifestyle modification, COVID-19 pandemic

## Abstract

Healthcare visits were reduced during the COVID-19 pandemic, causing disturbances in sustainable MAFLD monitoring. Telemedicine acts to maintain connectivity between patients and healthcare professionals. This review aimed to assess the role of telemedicine in monitoring MAFLD during the pandemic. Databases searched included *l* PubMed Central and ScienceDirect from 2020 to 2023. Assessment with The Cochrane Risk of Bias for randomized controlled trials (RCTs) and the Newcastle-Ottawa scale for non-RCTs systematic reviews. Meta-analyses employing a random-effect model were performed to determine the pooled mean difference (MD) and *p*-value. The results showed three RCT and two non-RCT (*n* = 239) with 56.9% males and a mean age of 51.3 years. The median intervention duration was 5.5 months. The parameters assessed included body weight (BW), body mass index (BMI), waist circumference, liver function (AST/ALT), lipid profile, HbA1c, and others. Meta-analysis revealed that telemedicine had a significant effect on improving outcomes for BW (MD −2.81: 95% CI, −4.11, −1.51, *p* < 0.0001) and BMI (MD −1.01: 95% CI, −1.47, −0.55, *p* < 0.0001) compared to standard care, while the AST/ALT levels were not significantly reduced. Some biochemical markers decreased based on the systematic reviews. In conclusion, telemedicine using mobile-based applications could be an option for monitoring lifestyle modification in MAFLD patients facing the COVID-19 pandemic.

## 1. Introduction

Telemedicine utilizing mobile-based applications provides integrated health services that connect health workers and patients using virtual communication technology [1]. Before the pandemic era, telemedicine had already been implemented for counseling in the United States. However, both users and providers of telemedicine face numerous obstacles. A huge number of users utilized telemedicine inconsistently, causing a lack of sustainability in medical treatment. Providers encountered challenges, including costly investments required for privacy regulation of patient data and difficulties obtaining approvals from both local and national governments [2]. However, the outbreak of SARS-CoV-2 presented an opportunity for telemedicine to optimize communication between health workers and patients [1,2]. Government policy is necessary to regulate telemedicine so that comprehensive health services can be provided [2].

Approximately 272.5 million people worldwide have been infected with severe acute respiratory syndrome coronavirus 2 (SARS-CoV-2), resulting in approximately 5.3 million deaths due to coronavirus disease 2019 (COVID-19) [3]. This RNA virus belongs to the Coronaviridae family. It interacts with the angiotensin-converting enzyme-2 (ACE-2) binding site on host cells in the heart, lungs, kidneys, and gastrointestinal tract. The mode of transmission of SARS-CoV-2 involves airborne infectious particles and aerosols, which are transmitted from infected individuals to their close contacts [4,5].

Metabolic dysfunction-associated fatty liver disease (MAFLD), previously known as non-alcoholic fatty liver disease (NAFLD), is a chronic liver disease characterized by excessive fat accumulation in the liver, without another obvious cause such as no excessive alcohol consumption, hepatotoxic medications, toxins, viral infections, genetic hepatic diseases [6,7]. MAFLD is defined as the accumulation of hepatic steatosis, as determined using imaging and/or liver biopsy and accompanied by at least one of three conditions, namely type 2 diabetes mellitus (T2DM), obesity, and metabolic dysregulation that can happen in both developed and developing countries [6,7,8]. The updated clinical guidelines from the American Association for the Research of Liver Diseases (AASLD), the American College of Gastroenterology, and the American Gastroenterological Association stipulate that the diagnosis of MAFLD should be based on three criteria: (1) evidence of increased hepatic fat on imaging, (2) absence of significant alcohol consumption (>30 g/day), and (3) absence of other known causes of chronic liver disease [9,10].

Typically, individuals with MAFLD remain asymptomatic. However, this condition can potentially progress to an end-stage state due to complex liver-cell injury and the accumulation of inflammatory cells, leading to a transformation of normal histology into a more aggressive form called metabolic dysfunction-associated steatohepatitis (MASH). MASH, in turn, can gradually evolve into the formation of fibrous scars characterized by varying degrees of fibrosis and will become liver cirrhosis. This situation will elevate the risk of morbidity and mortality among MAFLD patients due to liver cancer by approximately 1–2% annually in the absence of tailored and personalized treatment for each individual patient [6,8].

The current approach to treating MAFLD starts with identifying and addressing comorbidities according to established guidelines and implementing lifestyle modifications such as dietary changes, weight loss, and increased physical activity. These lifestyle adjustments serve as the cornerstone of MAFLD treatment [11,12,13]. Specifically, no medication has been approved as the primary treatment option for the disease [12].

Meanwhile, comorbidities such as obesity, diabetes, and fatty liver are associated with an elevated risk of developing severe COVID-19 [14,15]. Recent studies have demonstrated that individuals with MAFLD are at a four to six times higher risk of experiencing a worsening of their respiratory signs and symptoms compared to those who do not have fatty liver disease [15]. A study carried out in China discovered that severe COVID-19 was present in 70 out of 324 patients with fatty liver identified via abdominal computed tomography (CT) scans [16]. Nonetheless, patients with comorbidities were more likely to have compromised immunity, which facilitated robust viral replication. The SARS-CoV-2 virus may directly infiltrate hepatocytes via angiotensin-converting enzyme 2 (ACE2) located on the cytomembrane of hepatocytes. This process induces excessive activation of pro-inflammatory cytokines and exacerbates the cytokine storm associated with COVID-19. The viral and inflammatory processes might infiltrate hepatocytes, leading to hepatocyte damage. This cascade can elevate clinical biomarkers of hepatocyte injury, such as alanine aminotransferase (ALT), aspartate aminotransferase (AST), and bilirubin [17,18].

AST/ALT are essential diagnostic biomarkers for evaluating liver disease and abnormalities [18]. According to a multicenter study conducted in ten different cities in China, 30.7% (*n* = 103) of patients with COVID-19 and MAFLD exhibited elevated levels of AST and ALT [16]. According to a study conducted at a single site in India, which investigated the outcomes of COVID-19 infection in patients with or without MAFLD, the serum levels of ALT and AST were found to be significantly higher in patients with MAFLD [19].

Due to the COVID-19 pandemic, there were limitations on patients accessing healthcare facilities and restrictions on outdoor movement and exercise [20,21]. However, advancements in telemedicine have significantly progressed to face this pandemic era, offering a solution to monitor patients when social distancing is required. The impact of monitoring patients using telemedicine has enhanced the overall quality of medical services, encompassing both communicable diseases (such as COVID-19) and non-communicable diseases (such as asthma, hypertension, diabetes, etc.) [1,22]. During the pandemic, telemedicine could assist hepatologists in delivering healthcare for lifestyle modification in fatty liver disease, with a majority of patients expressing satisfaction with this approach [23]. This also could serve as a bridge to integrate standardized telemedicine into regular standard care delivery [1,22,23]. 

Therefore, the objective of this review was to assess how telemedicine could aid in monitoring lifestyle modification as a treatment of MAFLD patients during the COVID-19 pandemic, albeit focusing solely on MAFLD patients without coinfection with COVID-19.

## 2. Materials and Methods

This article was conducted in accordance with the Preferred Reporting Items for Systematic Reviews and Meta-Analyses (PRISMA) statement.

### 2.1. Search Strategy

A comprehensive search strategy was employed, utilizing literature databases such as PubMed Central and ScienceDirect, covering the period from June 2020 to July 2023. Manual searches were also conducted in JMIR mHealth and uHealth to enhance the comprehensiveness of the search strategy further. The search strategy is used keywords to retrieve articles, including “telemedicine”, “telehealth”, “telemessaging”, or “digital health intervention”, “non-alcoholic fatty liver disease”, “NAFLD”, MAFLD or “hepatic steatosis”, and “weight loss”, “lifestyle modification”, or “lifestyle changes”, along with “liver function test” and “biochemical markers.” The following results were exported to Zotero 6.0.26 to identify and manage duplicate records.

### 2.2. Research Selection and Data Extraction

The inclusion criteria for this literature review were as follows: (1) original research from either randomized controlled trials (RCTs) or non-RCTs; (2) studies involving human subjects; (3) publication between 2020 and 2023; and (4) availability of English language and online access, with full-text articles freely accessible. There were no limitations on patient criteria for inclusion in this review. Two independent reviewers, Safira Rosiana Choirida and Ahmad Zaqi Zaenal Muttaqin, screened the titles and abstracts of the relevant studies and reviewed the full text of the selected studies; a third and fourth reviewer, Femmy Nurul Akbar and Hari Hendarto, reviewed the full text of the selected studies and discussed discrepancy. The collected journal articles were also transferred to Microsoft Excel 2019 for data extraction. This facilitated the organization of specific results, including authorship, year of publication, design, setting, sample size, intervention methods, health worker related, and outcomes.

### 2.3. Risk of Bias Assessment

Two independent reviewers, Safira Rosiana Choirida and Ahmad Zaqi Zaenal Muttaqin, assessed the risk of bias using the Cochrane risk-of-bias tool for randomized controlled trials (RCTs) and the Newcastle-Ottawa scale (NOS) for non-randomized controlled trials (non-RCTs) [24,25]. Discrepancies in the results were resolved by the third and fourth reviewers (Femmy Nurul Akbar and Hari Hendarto).

### 2.4. Data Analysis

**Figure 1:**
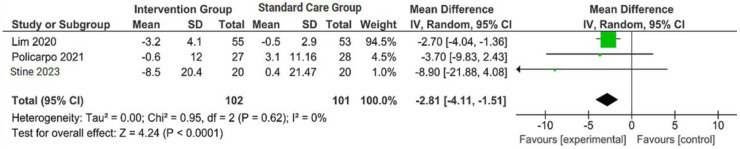
Forest plot of the effects of digital health intervention group compared with standard care group on the body weight (BW) [26,27,28]. Green square: results of individual studies effect, green dot: weight given in each study, black arrow: represent the 95% confidence interval (CI), black rhombus: overall summary effect.

**Figure 2:**
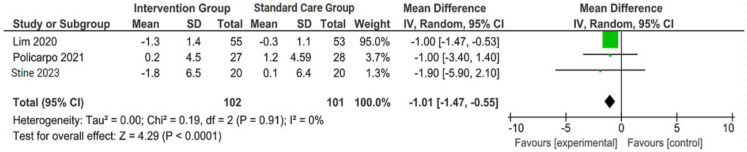
Forest plot of the effects of digital health intervention group compared with standard care group on the body mass index (BMI) [26,27,28]. Green square: results of individual studies effect, green dot: weight given in each study, black rhombus: overall summary effect.

**Figure 3:**
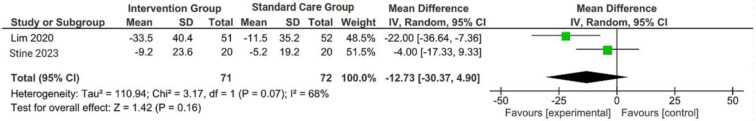
Forest plot of the effects of digital health intervention group compared with standard care group on the alanine aminotransferase (ALT) [26,28]. Green square: results of individual studies effect, black rhombus: overall summary effect.

**Figure 4:**
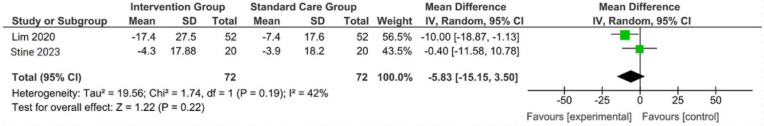
Forest plot of the effects of digital health intervention group compared with standard care group on the aspartate aminotransferase (AST) [26,28]. Green square: results of individual studies effect, black rhombus: overall summary effect.

The outcomes were reported as quantifiable measures to evaluate the effect of the intervention on lifestyle modification concerning body mass index (BMI), body weight (BW), liver function indicators such as alanine aminotransferase (ALT) and aspartate aminotransferase (AST), waist circumference (WC), lipid profile, and hemoglobin A1c (HbA1c). Furthermore, a qualitative analysis was conducted by extracting the data using Microsoft Excel to create descriptive text and tables and analyzing the outcomes of all parameters. For the meta-analysis, studies that did not perform standard care for the comparison with the intervention group were excluded. The analysis was carried out using Review Manager software (version 5.4, Cochrane Collaboration, 2020). The mean, standard deviation (SD), sample size of the intervention, and standard care group were inputted, and mean difference (MD) with 95% confidence interval (CI), while statistical heterogeneity using the I^2^ statistic. Finally, the illustrated results of the metanalysis were visually presented in a forest plot (Figure 1, Figure 2, Figure 3 and Figure 4).

## 3. Results

### 3.1. Research Selection

Out of the initial 90 studies sourced from databases, 88 studies were retrieved from PubMed Central and ScienceDirect. Additionally, two studies were manually added from JMIR, Mhealth, and Uhealth to maximize the tracing. After the removal of duplicates and application of filters, followed by screening for full-text eligibility screening, nine articles were prepared for manual screening. After excluding four articles that did not use telemedicine-based intervention, we included five studies as systematic reviews and meta-analyses and added 29 articles to explain this idea of the research.

**Figure 5:**
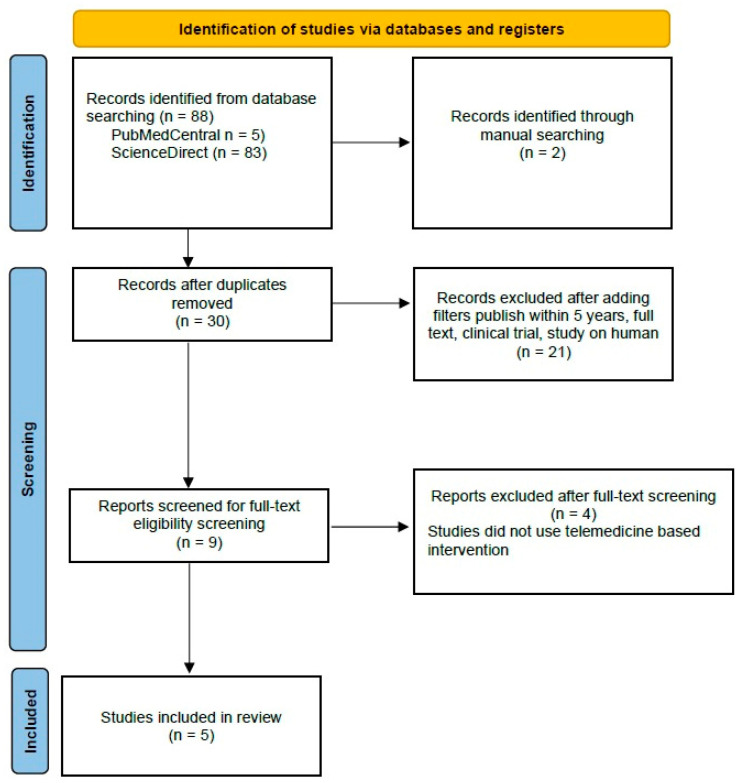
PRISMA Flow Chart of Literature Search.

PRISMA (Preferred Reporting Items for Systematic Reviews and Meta-Analyses) Flow Chart is depicted in Figure 5. Four studies were identified that reported data on body weight (BW), body mass index (BMI), alanine aminotransferase (ALT), and aspartate aminotransferase (AST).

### 3.2. Research Characteristics

The characteristics of the included studies were presented in Table 1, supplemented with additional information. Predominantly, the studies were conducted in the USA [26,29,30], followed by Portugal [27] and Singapore [31]. Three out of the five studies, namely Lim et al., Policarpo et al., and Stine et al., were randomized controlled trials (RCTs) conducted at a single center within outpatient hospital and clinic settings. The remaining non-RCT studies were conducted by Motz et al. and Tincopa et al. The total sample size consisted of 239 patients, divided into intervention and standard care groups, with the exception of Motz et al. and Tincopa et al., who did not provide information on standard care. Overall, 136 (56.9%) males and 103 (43.1%) females were recruited; Motz et al. exclusively recruited female participants due to COVID-19 restrictions. The majority of participants were obese patients of Caucasian descent, followed by Chinese individuals. The mean age of the participants was 51.3 years. The median duration of the intervention was 5.5 months. Most studies indicated their adherence to inclusion criteria in line with recent guidelines of MAFLD. Additionally, all studies shared the same objective of assessing the potential benefits of telemedicine for facilitating lifestyle changes among patients with MAFLD.

### 3.3. Assessment of Bias

Only RCT studies were considered for inclusion in the Cochrane Risk of Bias (RoB) tool assessment [25]. Among these three studies, Lim et al. reported the highest risk of detection bias, as they mentioned that the assessors were not blinded to the study-allocated groups. Policarpo et al. and Stine et al. did not clearly specify whether participants and personnel were blinded (performance bias) or how detection bias was addressed.

**Figure 6:**
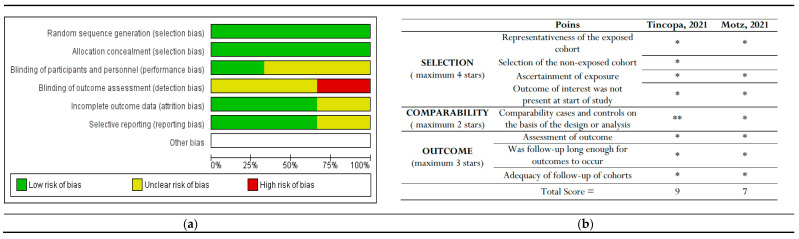
The risk of bias graph displays the review authors’ assessments of each risk of bias item presented as percentages across all included studies. This includes (**a**) Cochrane risk-of-bias assessment for randomized controlled trials (RCTs) and (**b**) New-castle Ottawa risk of bias assessment for non-RCT studies [29,30]. * and ** : indicate the rating for these categories.

The bias assessment outcomes for non-RCTs indicated that Tincopa et al. achieved the most favorable outcomes. The risk-of-bias assessments are depicted in Figure 6.

### 3.4. Meta-Analysis: The Pooled Effects of Telemedicine

Out of the five included studies, three were included in the meta-analysis, while two studies were conducted with single-arm designs. Three studies (Lim et al., Policarpo et al., Stine et al.) reported outcomes regarding BW and BMI [26,31]. However, one study (Policarpo et al.) did not provide the standard deviation outcomes for alanine aminotransferase (ALT) and aspartate aminotransferase (AST) [27].

A concise summary of the included studies was compiled into a forest plot illustrating body weight, BMI, ALT, and AST. Outcomes from the meta-analyses (Figure 1) suggested no heterogeneity in body weight and BMI (I^2^ = 0%). The pooled MD for the effects of digital health intervention on body weight (MD −2.81: 95% CI, −4.11 to −1.51, *p* = 0.0001) and BMI (MD −1.01: 95% CI, −1.47 to −0.55, *p* = 0.0001) were statistically significant. However, no statistically significant relationship between ALT (MD −12.73: 95% CI, −30.37 to −4.90, *p* = 0.16) and AST (MD −5.83: 95% CI, −15.15 to −3.50, *p* = 0.22), with a moderate level of heterogeneity (I^2^ = 68% for ALT and I^2^ = 42% for AST).

### 3.5. Result of Individual Studies

All the included studies focused on lifestyle modifications, including weight loss, physical activity, and stress management. We identified four studies concerning BW, BMI, AST, and ALT. The mobile application provided access focusing on body weight loss and behavioral changes, which technically involved monitoring and providing personalized feedback to patients. Additionally, three studies addressed WC, while two studies investigated lipid profiles and HbA1c.

However, each study had its main topic, including lifestyle change programs. Stine et al. study focused on a lifestyle change program encompassing nutrition, physical activity, and sustainable behavioral changes facilitated using a mobile-based health application or telemedicine called NoomWeight (NW), while Lim et al. was composed of dietary and lifestyle guidance via telemedicine known as nBuddy [26,31]. Moreover, Tincopa et al., which employed the utilization of FitBit, a mobile digital health technology, had the objective of monitoring daily steps and providing nutritional assessments. Patients could easily log their daily meals, daily step counts, and body weight and access a wealth of health and stress management information [30]. In contrast, studies by Motz et al. and Policarpo et al. utilized video and/or phone communication as their primary digital health intervention for monitoring the intervention group (IG) [27,29].

In most of the included studies, participants were instructed to engage in physical activities, at least with moderate-intensity aerobic training. Regarding dietary intervention, three studies considered providing recommendations for the Mediterranean diet [26,27,29,30], one study represented nutritional counseling according to the original research protocol [29], and one study represented dietary intervention via general advice from a dietitian [31].

Stine et al. included 40 participants in their study, equally divided between an intervention group and a standard care group. The trial lasted for four months, during which both groups received education from a hepatologist regarding the current guidelines for MASH in clinical practice, focusing on the Mediterranean diet and 150 min of moderate-intensity physical activity per week. The IG received a dietary and physical activity program via the NoomWeight application. At the end of the trials, the IG exhibited a significant decrease in body weight (*p* = 0.0008) and BMI (*p* = 0.037) compared to the standard care (SC) group. Moreover, 45% of the patients successfully reduced 5% of their body weight. The IG also received a significant reduction in platelet count compared to SC (−28 vs. −5.7 × 109, *p* = 0.038). Other laboratory parameters were also evaluated, such as liver function test, albumin level, blood sugar level, and Fibrosis-4 index (FIB-4), but the results were not statistically significant, respectively (*p* > 0.05). Furthermore, 70% and 75% of IG met the criteria for feasibility and acceptability concerning the use of NoomWeight [26]. Stine et al. stated 43% of patients had stage F0/F1, and 40% had stage F2.

In a study conducted by Lim et al., participants underwent a 6-month RCT where the IG received guidance on dietary choices and physical activity from a dietitian using nBuddy mobile application to record their dietary intake, physical activity, and behavioral modifications while the SC group received guidance from a trained nurse at the clinic. All groups received dietary programs based on the guidelines provided by the American Heart Association. At the end of the trial period, the IG demonstrated notable reductions in body weight (mean 3.2, SD 4.1 kg vs. mean 0.5, SD 2.9 kg; *p* < 0.001), waist circumference (mean 2.9, SD 5.0 cm vs. mean −0.7, SD 4.4 cm; *p* < 0.001), systolic blood pressure (mean 12.4, SD 14.8 mmHg vs. mean 2.4, SD 12.4 mmHg; *p* = 0.003), diastolic blood pressure (mean 6.8, SD 8.9 mmHg vs. mean −0.9, SD 10.0 mmHg; *p* = 0.001), ALT (mean 33.5, SD 40.4 IU/L vs. mean 11.5, SD 35.2 IU/L; *p* = 0.004), and AST (mean 17.4, SD 27.5 U/L vs. mean 7.4, SD 17.6 IU/L, *p* = 0.03) compared to SC group [31].

Policarpo et al. conducted a study using NAFLD-HIV patients from an outpatient infectious disease clinic assigned to the IG or the SC group. Both groups received uniform guidance pertaining to a structured dietary intervention, emphasizing caloric restriction and weight loss strategies rooted in the Mediterranean diet. During the 3rd and 4th months of the research, patients underwent a review of dietary advice, eating habits, and lifestyle modifications during the pandemic via video and/or phone calls, which also included the completion of four stress questionnaires. Before the implementation of the lockdown, the IG exhibited a decrease in body weight, with a median loss of 1.5 kg compared to a median loss of 0.65 kg in the Standard Care (SC) group (*p* < 0.001). Following 3 months of lockdown, both groups experienced weight gain, with the SC group showing a higher weight gain of around 3 kg compared to less than 1 kg in the IG (*p* < 0.001) [27]. Policarpo et al. also presented that 63.6% of patients had no evidence of fibrosis, 27.3% of patients had a moderate degree of fibrosis (F2–F3), and 9.1% had severe fibrosis (F4) using Liver transient elastography (Fibroscan©, Echosense, France) that underwent before intervention [26]. However, the researcher could not perform a Fibroscan after intervention due to local and government issues.

Some studies used the Fibrosis-4 (FIB-4) index or NAFLD fibrosis score (NFS), which is also used to predict the severity of fibrosis in MALFD patients. FIB-4 evaluated the risk of liver fibrosis by calculating on the basis of age, AST/ALT levels, and platelet count. Moreover, NFS predicted the severity of fibrosis based on six variables: age (years), BMI (kg/m^2^), fasting blood glucose, AST/ALT ratio, platelet count, and albumin levels. As the results, Policarpo et al. presented a low probability for liver fibrosis FIB-4 in 87.3% of patients [27], while Stine et al. revealed no significant changes in clinical scoring such as NFS and FIB-4 (*p* > 0.05) after intervention [26].

Motz et al. conducted a study involving 20 weeks of moderate-intensity aerobic training delivered via telehealth, which was administered to patients with non-alcoholic fatty liver disease (NAFLD) under direct supervision using an audio-visual telehealth platform. Body weight, body fat percentage, and waist circumference improved with exercise. Mean reduction was also shown in HbA1c levels, AST/ALT levels, and homeostatic model assessment for insulin resistance (HOMA-IR) with respective values. Subsequently, all participants met the priori definition of feasibility [29].

Furthermore, Tincopa et al. also illustrated the implementation of physical activity utilizing the FitBit mobile application and conducted nutritional assessments to evaluate the feasibility and acceptability of lifestyle modifications among NAFLD patients using mobile technology. They intervened with the participants to assess secondary outcomes, encompassing body weight, physical fitness, liver transient elastography, laboratory testing, and quality of life. They had statistically significant improvements in waist circumference lipid profile parameters, including HDL, LDL, and triglycerides, along with reductions in hemoglobin A1c levels, with a *p*-value of < 0.05. Approximately 50% of the participants exhibited reductions in BW and ALT and a 42.4% reduction in liver stiffness or fibrosis by liver transient elastography (Fibroscan). Roughly 59% of the participants reported that the mobile application was easy to use, and 66% of the patients expressed motivation to enhance their physical activity while utilizing the daily step count tracker. These outcomes suggest that these tools are not only feasible but also acceptable and hold promise for future interventions [30].

## 4. Discussion

The COVID-19 pandemic has imposed limitations on patients accessing healthcare facilities, leading to a decrease in treatment sustainability. One breakthrough is the utilization of telemedicine to enhance connectivity between patients and healthcare professionals. Lifestyle modification remains the cornerstone of treatment for MAFLD due to insufficient evidence supporting pharmacological interventions [6,12].

This systematic review summarizes the usage of telemedicine for monitoring lifestyle modification interventions in adults with MAFLD. Most participants were males, similar to the incidence of fatty liver disease globally that males are higher than females, although one included study exclusively recruited females [29]. The mean age was 51.3 years, similar to the most prevalent worldwide, which was 51–60 years. All included studies were published between 2020 and 2023.

Multiple guidelines have indicated that achieving a reduction in body weight of 3–5% could lead to improvement in hepatic steatosis, while a reduction of 7% in body weight could induce changes in the histological features of metabolic-associated steatohepatitis (MASH) [16]. For MAFLD, dietary recommendations include reducing daily calorie intake by 500–1000 kcal per day (hypocaloric diet), adherence to the Mediterranean diet, adoption of a low-carbohydrate diet, and adherence to a low-fructose diet. Additionally, moderate-intensity aerobic physical activity with a minimum duration of 150–200 min per week or 30 min per day and a frequency of more than twice per week (3–5 days a week) is recommended [12].

Based on our meta-analysis, the intervention group employing telemedicine was significantly more effective in achieving reductions in body weight and BMI compared to standard care in MAFLD patients. It is also observed from all included studies that reducing body weight is associated with a subsequent decrease in biochemical markers such as AST and ALT. However, this meta-analysis revealed that telemedicine did not significantly decrease AST and ALT levels. Waist circumference was reported in three studies, indicating improvement in the intervention groups. One study showed improvements in other markers of MAFLD risk factors, including a panel of lipids, liver stiffness, and HbA1c, but they were not statistically significant [30]. Other studies showed improvement in blood pressure systolic as well as diastolic pressure [31]. Motz’s study also revealed improvement in` BW, BMI, waist circumference by exercise, and a mean reduction in AST/ALT, HbA1c, and HOMA-IR with respective values.

The degree of liver fibrosis was assessed in three studies using liver transient elastography (Fibroscan) [26,27,30,31]. It was concluded that most of the degrees of fibrosis in the included studies had no evidence of mild fibrosis (F0/F1), and there is no study that showed statistically significant improvement in liver stiffness or fibrosis by Fibroscan [27,30]. FIB-4 index and NFS also showed no clinically significant changes after intervention [26].

Based on outcomes, the duration and frequency of digital interventions varied widely, ranging from 4 to 6 months, but the majority of articles implemented interventions lasting 5–6 months. At present, there are no established standard regulations regarding the duration of digital interventions in MAFLD patients and their potential impact on the clinical and biochemical outcomes of MAFLD. However, the included studies showed that reducing body weight by 5% was found to be more effective when the intervention was implemented for more than 4 or 6 months [29,30,31]. The changes in biochemical markers were also notably reduced over a duration of 5–6 months [28,29]. Hence, the intervention for 5–6 months was found to be more sustainable.

Telemedicine serves as a bridge that facilitates health workers, including medical students, in monitoring patients indirectly and virtually. Additionally, there were advantages for patients in terms of cost savings, as they could avoid visiting onsite healthcare facilities [28,32]. This demonstrates that telemedicine is both feasible and acceptable for monitoring patients with MAFLD, primarily via the use of mobile applications [33,34,35].

The role of telemedicine may commence with primary healthcare practitioners for early detection using risk factors, progressing to further evaluation and categorizing patients into risk groups for referral to higher-level healthcare facilities. If the patient is deemed to have a low risk of disease progression, they will be referred back to their primary healthcare for lifestyle modification and to receive ongoing laboratory monitoring. Meanwhile, patients in the intermediate and high-risk stages of disease progression will be referred to tertiary healthcare for monitoring and therapy. Additionally, they will receive lifestyle modification guidance from primary healthcare providers using telemedicine [23,33,34,35].

The limitation of our study resided in the relatively small number of included studies and participants, as well as the fact that the majority of studies were conducted in Western countries (3 in the USA, 1 in Portugal, and 1 in Singapore). Hence, the interventions in those studies remain applicable in real-world settings. Another limitation of the included studies was the variability in the competencies of the workers involved in making recommendations or implementing interventions.

Our study strengths in its focus solely on current studies conducted during the pandemic, ensuring its up-to-dateness. Therefore, despite the challenges in real-world settings associated with its implementation, telemedicine could be utilized for monitoring body weight and BMI, given the presence of adequate facilities, infrastructure, and government support.

## 5. Conclusions

This systematic review and meta-analysis revealed that telemedicine is beneficial in reducing body weight and BMI in patients with metabolic dysfunction-associated fatty liver disease (MAFLD). However, our meta-analysis indicated that telemedicine did not significantly lower liver function markers such as AST and ALT levels in MAFLD patients. Additionally, waist circumference was found to be lower in the intervention groups across three studies. Moreover, other markers associated with MAFLD risk factors, such as a panel of lipids and HbA1c levels. Telemedicine has the potential to facilitate healthcare professionals in remotely monitoring patients, including tracking their body weight, dietary intake, daily physical activities, and biochemical markers. Patients could save money by avoiding visits to healthcare facilities. This demonstrates the feasibility and acceptability of telemedicine for monitoring patients with MAFLD, particularly using mobile applications. Telemedicine enables patients to maintain connectivity to healthcare professionals while mitigating the risks of disease exposure, including the transmission of COVID-19 infection in traditional offline healthcare settings.

In conclusion, telemedicine could serve as a viable option for monitoring lifestyle modification, including body weight and BMI in MAFLD patients amidst the COVID-19 pandemic, and offer the patients convenience to remain connected with their healthcare providers while preventing the spread of COVID-19 infection. Future studies should be conducted on larger populations to assess the generalizability of telemedicine utilization in clinical settings.

## Figures and Tables

**Table 1:** Summary of the included studies.

Author, Year, Country.	Setting	Sample Size	Dur. (Mon.)	Group		Types of Telemedicine	The Health Coworker Related	Outcome	Outcomes
				Intervention	Control				
Lim SL, 2020, [28] Singapore RCT, single center	National University Hospital Singapore	108 adults 55 IG, 53 SC	6	Patients underwent private consultations offline with a dietitian, followed by online consultations via a mobile app (nBuddy). They were instructed on how to utilize the app to log and track their food intake, physical activity, and behavioral changes.	Standard dietary and physical activity recommendations based on AHA guidelines	Mobile telehealth (nBuddy)	Single dietician, trained nurse, assessors.	BMI, BW, AST, ALT, WC, SBP, DBP	BMI *, BW *, AST *, ALT *, WC *, SBP *, DBP *
Motz V, 2021 [29] Non-RCT, single center	RCT patients	3 adults of IG	5	Moderate-intensity aerobic exercise for a duration of 30 min, conducted on 5 days per week.	NA	Direct supervision using audio-visual telehealth platform	Exercise physiologist	BMI, BW, ALT, AST, WC, HbA1c	BMI #, BW #, ALT # AST #, WC #, HbA1C, Homeostasis model assessment for insulin resistance (HOMA-IR)3
Tincopa, 2021, USA [30] Non—RCT, single-center	General hepatology outpatient clinic	40 adults of IG	6	Participants are required to engage in physical activity via walking or step counting, with a target range of a minimum of 800 steps per week to 10,000 steps per week. Additionally, personalized feedback on physical activity is provided.	NA	FitBit mobile application, phone call	The researchers	BMI, BW, ALT, AST, WC, SBP, Lipid Profile, HbA1c	ALT *, WC *, lipid profile *, HbA1c * BW ^, ALT ^ Fibroscan ^
Policarpo S, 2021, Portugal [27] RCT, single-cener.	Outpatients Infectious Disease Clinic	55 adults 27 IG, 28 SC	6	The nutritional plan is founded on the Mediterranean diet and is provided in the format of written healthy eating guidelines and tips. It entails a reduction of 500 kilocalories in daily calorie intake, abstaining from alcohol consumption, and limiting fruit consumption to less than 5 units per day.	General dietary recommendations	Video and/or phone	Single dietician	BMI, BW, Lipid profile, Blood glucose Liver stiffness (Fibroscan)	BMI *, BW *, Lipid profile Blood glucose, FIB-4 index Fibroscan ^$^
Stine JG, 2022, USA [26] RCT, single-center	Penn State Milton S. Hershey Medical Center and the Penn State College of Medicine	40 adults 20 IG, 20 SC	4	Human coaching from an academic hepatologist regarding the Mediterranean diet and moderate-intensity physical activity, with a minimum of 150 min per week. NoomWeight’s curriculum	General dietary recommendations	Mobile application	Hepatologist	BMI, BW, Albumin, ALT, AST, fasting blood glucose Platelet, FIB-4, HbA1c	BMI *, BW *, Platelet * Albumin, ALT, AST, ALP, FIB-4 index, NFS, fasting glucose. HbA1c

IG: intervention group, SC: standard care, MAFLD: metabolic dysfunction-associated fatty liver disease, BMI: body mass index, BW: body weight, AST: aspartate aminotransferase, ALT: alanine aminotransferase, WC: weight circumference, SDP: systolic blood pressure, DBP: diastolic blood pressure, FIB: fibrosis-4 index, HbA1c: hemoglobin A1c, RCT: randomized controlled trial, AHA: American Heart Association, ALP: alkali phosphatase, NFS; NAFLD fibrosis score. *: the outcomes of the studies were significant, #: significant without *p*-value, ^: reduced but not significant, ^$^: cannot be done after intervention.

## Data Availability

The data depicted in this research are obtainable upon request from the corresponding author.
